# Cardiopulmonary Bypass Ischemic Hepatitis Reported in Five Patients

**DOI:** 10.5935/1678-9741.20160059

**Published:** 2016

**Authors:** Telma A. Damasceno, Adilson Scorzoni, Fernando Chahud, Alfredo José Rodrigues, Walter Vilella de Andrade Vicente, Paulo Roberto Barbosa Evora

**Affiliations:** 1 Division of Cardiovascular and Thoracic Surgery, Department of Surgery and Anatomy, Faculdade de Medicina de Ribeirão Preto da Universidade de São Paulo (FMRP-USP), SP, Brazil.

**Keywords:** Cardiopulmonary Bypass, Liver Failure, Hepatitis

## Abstract

**Objective:**

In cardiac surgery, the lung, renal and neurological events are the most frequent complications. Less common, acute liver failure is a serious complication that adds high morbidity, mortality, and costs. Therefore, this communication aimed to retrospectively evaluate five patients who presented, in 2014, severe acute liver failure in the immediate postoperative period.

**Methods:**

Retrospective data analysis of patients' medical records that showed severe liver failure has been computed in the medical records of five patients undergoing cardiac surgery at the Hospital da Faculdade de Medicina de Ribeirão Preto – USP in the immediate postoperative period from February 1, 2014 to December 12, 2014. The study selected five males patients, 60 to 67 years old, cardiopulmonary bypass mean time of 101.4 minutes (varying from 80 to 140 minutes), who presented acute perioperative liver failure.

**Results:**

The five patients showed an impressive increase of blood transaminase (serum alanine aminotransferase), suggesting acute hepatitis. The evolution of all patients was catastrophic, with severe hemodynamic effects and death. Many studies suggest that systemic hypotension is an important pathogenic factor for ischemic hepatitis. However, our data and previous studies raise the possibility that other yet unknown factors other than hypotension may be part of the pathophysiology of cardiopulmonary bypass after ischemic hepatitis (anticoagulation inadequate for the quality of heparin and protamine, etc.).

**Conclusion:**

Currently, there are no conclusive studies on the prevention of perioperative liver failure. More well-designed studies are needed on the introduction and evolution of liver dysfunction after cardiac surgery.

**Table:** 

Abbreviations, acronyms & symbols	
CI	= Cardiac index
CPB	= Cardiopulmonar bypass
CVP	= Central venous pressure
LDH	= Lactate dehydrogenase
LFTs	= Liver function tests
NYHA	= New York Heart Association
SIELI	= Severe ischemic early liver injury

## INTRODUCTION

Cardiopulmonary bypass (CPB) is closely related to the progress of cardiac surgery in complexity. However, it is well known that the postoperative complications are directly related to the preoperative patients comorbidities and prolonged CPB time. The most common ones are lung, kidney, and neurological complications. The less common one, acute liver failure, is a serious complication that adds high morbidity, mortality, and costs^[1]^. Instead ischemic hepatitis, some authors prefer to use an unspecific nomenclature suggesting and adopting severe ischemic early (first 48 hours) liver injury (SIELI) after cardiac surgery is considered a rare postoperative complication with a limited mention in the literature^[2,3]^. As ischemic hepatitis is considered of low incidence, the study rationale included the intriguing observation of five cases in a short time interval (ten months). Therefore, the aim of this investigation was to identify patients with liver failure, classified as ischemic hepatitis, in the immediate postoperative period when undergoing cardiac surgery.

## PATIENTS

The Institutional Ethics Committee waived the need for informed consent for this anonymous retrospective chart analysis. The study included a cohort of five males patients, with ages ranging 60-67 years, with CPB time varying from 80 to 140 minutes (mean time of 101.4 minutes) (Table 1), who showed acute liver failure after CPB. For the purpose of this study, SIELI was defined as an acute increase in serum alanine aminotransferase levels to more than 500 IU/L within 48 hours of surgery. In all patients, induction and maintenance of anesthesia were performed according to the standard protocol, using midazolam, sufentanil, and vecuronium. Only one patient received inhaled sevoflurane, and other drugs are presented in Table 2. CPB was maintained according to an alpha-stat protocol. In blood gas management, the arterial pH was maintained at 7.4, and the arterial PCO_2_ was regulated from 35 to 40 mmHg. Total CPB mean flow was 3.77 L/min/m^2^ (0.8 to 5.7 L/min/m^2^) and mean arterial pressure during bypass was kept at greater than 70 mmHg with the use of norepinephrine if necessary. Minimal core body temperature was 33°C.

**Table 1:** Demographic and surgery data.

Name	Age	Gender	Ejection Fraction	Surgery Date	Surgery	CPB time (min)	Anoxia time (min)
JTR	66	M	62%	12/17/2014	CABG	102	87
JJG	67	M	23%	06/24/2014	CABG	95	49
SSR	63	M	45%	07/17/2014	MiVP	140	101
AGN	60	M	50%	02/13/2014	AoVP	90	65
AAB	60	M	33%	07/15/2014	AoVP	80	60

CPB=cardiopulmonary bypass; CABG=coronary artery bypass graft; MiVP=mitral valve prosthesis; AoVP=aortic valve prosthesis

**Table 2:** Intraoperative drugs.

	Name				
Drug (mg)	AGN	AAB	JJG	JTR	SS
Midazolam	5	5	10	9	5
Sulfentanil	150	65	50	220	45
Morfina	-	400	-	-	-
Etomidate	20	20	20	20	20
Vecuronium	5	8	50	10	10
Ketamine	-	-	-	-	-
Cefazolyn	3	-	-	-	3
Vancomycin	-	1000	1000	1000	-
Amicacyn	-	500	500	500	-

**Fig. 1:**
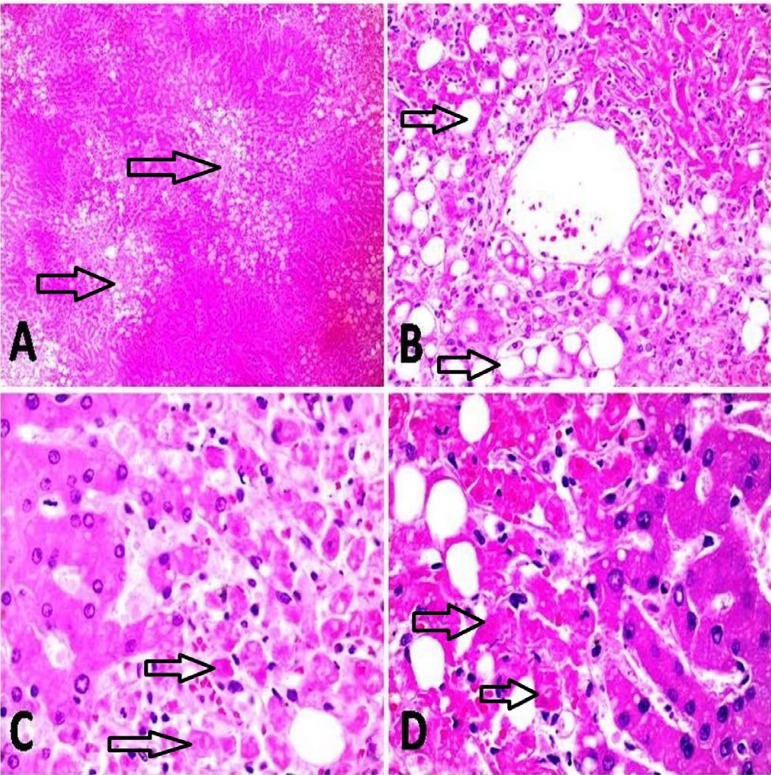
A – several hepatocellular foci of necrosis (arrows) and steatosis predominantly surrounding centrolobular veins; B – details of hepatocellular necrosis and steatosis (arrows) with many anucleated hepatocytes presenting eosinophilic, degenerating cytoplasms; C – interface between viable hepatocytes (on the left) and necrotic cells (on the right). The anucleated necrotic hepatocytes show contracted cytoplasms (arrows); D – interface between viable hepatocytesa (on the right) and necrotic cells (on the left). The anucleated necrotic hepatocytes show contracted cytoplasms (arrows).

All five patients presented an impressive increase of blood transaminase, respectively: 1022; 2027; 3624; 7359 and 2583 U (3383±2444.5 U), suggesting acute hepatitis. A post-mortem histological examination revealed centrilobular liver necrosis that is commonly misdiagnosed as ischemic hepatitis (Figure 1). Survival time in Intensive Care Unit was on average 5.4 days after surgery, ranging from 2 to 13 days. The evolution of all patients was catastrophic, with severe hemodynamic effects, multiple organ failures, and death (Table 3).

**Table 3:** Clinical laboratory data.

	Aspartate Transaminase (AST)		Glucose		Amylase
Name	Pre	Post	Pre	Post	Post
JTR	54.3	4119 (13^rd^ day)	57	-327	509 (1^st^ day)
JJG	29	4277.8 (2^nd^ day)	90	291	132 (2^nd^ day)
SSR	25.3	1022.3 (3^rd^ day)	37	194	458 (1^st^ day)
AGN	-	2027.6 (1^st^ day)	30	>249	-
AAB	14.2	2583.4 (4^rh^ day)	<69	>302	1017 (1^st^ day)

### DISCUSSION

The hepatic integrity is affected during cardiac surgery in particular when CPB is adopted^[4-7]^. The recent review performed by Di Tomaso et al.^[8]^ summarized the primary data about the severe liver dysfunction after CPB that can occur in high-risk patients with a reduced physiological reserve. Pre-operative risk factors are right-side heart failure, moderate-to-severe tricuspid regurgitation, pulmonary hypertension (systolic pulmonary pressure above 45 mmHg), high preload [central venous pressure (CVP) above 8 mmHg] chronic heart failure, New York Heart Association (NYHA) class II-IV and low ejection fraction^[8]^ are at a higher risk to develop liver dysfunction after CPB. Interestingly, as reported by Van Deursen et al.^[9]^, in 323 patients with heart failure, the hemodynamic profile can affect the liver function tests (LFTs). In fact, elevated LFTs mainly indicate a higher CVP, whereas only the presence of elevated aspartate aminotransferase, alanine aminotransferase or direct bilirubinemia may indicate a low cardiac index (CI)^[9]^.

Ischemic hepatitis can be found after a period of relatively profound hypotension and hemodynamic instability, and it is often associated with left ventricular dysfunction. The reduction in the hepatic blood flow leads to a consequent hypoxia/anoxia of hepatocytes histologically characterized by the centrilobular necrosis of zone 3 hepatocytes. Biochemical markers of ischemic hepatitis are an increase in serum aspartate transaminase and serum alanine transaminase ALT 10-20 times the standard value, a rise of lactate dehydrogenase (LDH), total bilirubin and a deficiency of hepatic coagulation factors with a consequent prolongation of prothrombin time, 1-3 days after the CPB injury. Usually, these biochemical indices return to normal within 5-10 days. If the hepatic biomarkers remain persistently high, and other organs are affected from the perioperative systemic hypoperfusion, multiorgan failure can occur, and it leads to death in the majority of cases.

Eipel et al.^[10]^ emphasize the well-known physiological concept that among parenchymal organs, blood flow to the liver is unique due to the dual supply from the portal vein and the hepatic artery. Knowledge of the mutual communication of both the hepatic artery and the portal vein is essential to understand hepatic physiology and pathophysiology. To distinguish the particular importance of each of these inflows in normal and abnormal states is still a challenging task and the subject of ongoing research. A central mechanism that controls and allows constancy of hepatic blood flow is the hepatic arterial buffer response. This buffer response is, surely, affected by the artificial CPB hemodynamics allowing the controversial hypothesis that the hepatic stasis should be more deleterious than the ischemia-reperfusion injury.

Many studies suggest that systemic hypotension is an important pathogenic factor for ischemic hepatitis. However, our data and previous studies raise the possibility that other as yet unknown factors other than hypotension may be part of the pathophysiology of CPB after ischemic hepatitis. Reinforcing this hypothesis, it is important to note that the occurrence of the cases occurred in a short time (some pathogen inoculation? Anesthesia drugs? Inadequate anticoagulation due to the quality of heparin and protamine, etc). Sufentanil was used in all patients, but the pharmacokinetics of phenylpiperidine opioids such as fentanyl, sufentanil, and remifentanil appear to be unaffected by hepatic disease^[11]^.

Halogenated inhalational anesthetics are associated with liver injury. Halothane, enflurane, isoflurane, and desflurane are metabolized through the metabolic pathway involving cytochrome P-450 2E1 (CYP2E1) and produce trifluoroacetate components; some of which may be immunogenic. The severity of hepatotoxicity is associated with the degree by which they undergo hepatic metabolism by this cytochrome. However, liver toxicity is highly unlikely from sevoflurane as is not metabolized to trifluoroacetyl compounds. Only one patient received inhalational sevoflurane. Although anesthesia-induced hepatitis is not a common occurrence, we must consider the association between this disorder and the use of halogenated anesthetics^[12]^.

Hepatotoxic complications of long-term oral amiodarone therapy have been well described; however, liver injury secondary to a parenteral infusion of amiodarone is uncommon, potentially fatal, and poorly understood. The hepatotoxicity is thought to result from the diluent polysorbate 80 and not the amiodarone itself. Theories suggest an allergic or immunologic response leading to alterations in the hepatocellular membrane while some propose that ischemia, not a drug reaction, is true to blame. The patients under mitral valve surgery used chronically preoperative amiodarone, which can induce severe acute hepatitis. However, there is sufficient evidence to support amiodarone-induced acute hepatotoxicity as a unique entity separate from ischemic hepatitis^[13]^.

Currently, there are no conclusive studies on the prevention of perioperative liver failure. More well-designed studies on the presentation and evolution of liver dysfunction after cardiac surgery are needed. However, on the balance of probability, based on wide literature search, it is more likely that this hepatic failure is related to some intraoperative event rather than to drugs including the volatile anesthetics. The possible connection among the hepatic failure herein described and the drugs administered during this patient's anesthesia, and CPB should be merely coincidental. Also, based on literature data, the systemic hypotension or shock alone did not lead to ischemic hepatitis in any patient. However, the great majority of patients with ischemic hepatitis had passive liver congestion, suggesting that right-sided heart failure, with resultant hepatic venous congestion, may predispose the liver to hepatic injury induced by a hypotensive event.

### Study Limitation

The article category limits the text in 1500 words. For this reason, we opted for a more detailed data presentation as tables to better characterization of the patients. Some of these results are not displayed as text, or even discussed, and the reader should report a careful observation of the contents from tables.

**Table:** 

Authors’ roles & responsibilities	
TAD	Conception and design study; analysis and/or data interpretation; manuscript writing or critical review of its content; final manuscript approval
ASF	Realization of operations and/or trials; final manuscript approval
FC	Analysis and/or data interpretation; final manuscript approval
AJR	Realization of operations and/or trials; final manuscript approval
WVAV	Final manuscript approval
PRBE	Conception and design study; analysis and/or data interpretation; manuscript writing or critical review of its content; final manuscript approval
